# O018. The increased flow pulsatility into cerebral arterial network may play a role in the pathogenic mechanism of migraine headache?

**DOI:** 10.1186/1129-2377-16-S1-A60

**Published:** 2015-09-28

**Authors:** Stefano Viola, Paolo Viola, Maria P Buongarzone, Mafalda Cipulli, Luisa Fiorelli, Pasquale Litterio

**Affiliations:** Department of Neurology, Headache Center, Vasto, CH Italy; Emergency Medical Service, Atessa, CH Italy

## Background

It is now well accepted that migraine headache is mediated by the increased sensitivity and ensuing activation of trigeminovascular nociceptive afferents that innervate the dura mater and their related blood vessels. One of the fundamental questions is to determine which processes actually play a role in promoting such condition.

## Aim

To evaluate whether patients with episodic migraine with (MA+) and without aura (MA-), during the interictal period of migraine, would have an increased flow pulsatility into cerebral arterial network and whether it would play a role in migraine headache.

## Methods

To evaluate the flow pulsatility into cerebral arterial network, we measured the time-delay in milliseconds (ms) between the R-wave of an electrocardiogram and the arterial pulse wave of cerebral microcirculation (R-APWCMtd) on the frontal cortex detected by near-infrared spectroscopy (NIRS) in 10 patients with MA+ (age 39.5±12.2 years), in 10 with MA- (age 40.3±10.2 years), according to the ICHD-3 criteria (2013), during the interictal period of migraine, and in 15 age, sex and height matched healthy control subjects.

## Results

The patients with migraine had a significantly longer R-APWCMtd than the control subjects *F*= 13.4, p < 0.001: MA+: + 38.3 ms; MA-: + 34.7 ms indicating an increased distensibility of the wall of the cerebral arterial network. In multiple regression analysis, R-APWCMtd was significantly associated with migraine (R^2^ = 0.50, p < 0.0001) but not with age, gender, height, migraine attack frequency and disease duration.

## Conclusions

The increased distensibility, reducing the impedance mismatch between aorta and first-generation arteries, leads to an increased flow pulsatility into intracranial dural meningeal vessels that may lead to a mechanical stimulation of the nociceptors that innervate the dural vasculature. This condition may play a role in promoting the sensitization of trigeminovascular afferents and sterile inflammation within the dura mater that are fundamental to the pathogenesis of migraine headache.

Written informed consent to publication was obtained from the patient(s).

